# Size effects resolve discrepancies in 40 years of work on low-temperature plasticity in olivine

**DOI:** 10.1126/sciadv.1701338

**Published:** 2017-09-13

**Authors:** Kathryn M. Kumamoto, Christopher A. Thom, David Wallis, Lars N. Hansen, David E. J. Armstrong, Jessica M. Warren, David L. Goldsby, Angus J. Wilkinson

**Affiliations:** 1 Department of Geological Sciences, Stanford University, Stanford, CA 94305, USA.; 2 Department of Earth and Environmental Science, University of Pennsylvania, Philadelphia, PA 19104, USA.; 3 Department of Earth Science, University of Oxford, Oxford, UK.; 4 Department of Materials, University of Oxford, Oxford, UK.; 5 Department of Geological Sciences, University of Delaware, Newark, DE 19716, USA.

## Abstract

The strength of olivine at low temperatures and high stresses in Earth’s lithospheric mantle exerts a critical control on many geodynamic processes, including lithospheric flexure and the formation of plate boundaries. Unfortunately, laboratory-derived values of the strength of olivine at lithospheric conditions are highly variable and significantly disagree with those inferred from geophysical observations. We demonstrate via nanoindentation that the strength of olivine depends on the length scale of deformation, with experiments on smaller volumes of material exhibiting larger yield stresses. This “size effect” resolves discrepancies among previous measurements of olivine strength using other techniques. It also corroborates the most recent flow law for olivine, which proposes a much weaker lithospheric mantle than previously estimated, thus bringing experimental measurements into closer alignment with geophysical constraints. Further implications include an increased difficulty of activating plasticity in cold, fine-grained shear zones and an impact on the evolution of fault surface roughness due to the size-dependent deformation of nanometer- to micrometer-sized asperities.

## Teaser

When deforming by low-temperature plasticity, the strength of the mantle mineral olivine is controlled by its grain size.

## INTRODUCTION

The strength of the lithospheric mantle, the relatively cold and rigid outer layer of the mantle, during deformation by low-temperature plasticity controls a range of geological phenomena, because the mantle comprises up to ~95% of tectonic plates. For instance, lithospheric-scale strain localization, a necessity for the formation and longevity of plate boundaries, is primarily accommodated by plastic deformation of olivine (*1*), the dominant mineral of the upper mantle. Low-temperature plasticity of olivine is also critical in lithospheric flexure beneath volcanic islands (*2*) and at subduction zones (*3*), the evolution of friction on seismogenic faults (*4*), and subcritical crack growth in the mantle (*5*).

Experimentally derived equations that describe olivine plasticity are extremely variable in the strain rates they predict when extrapolated to geological conditions, especially at low temperatures. Estimates of olivine strength at room temperature, for example, vary between 2 and 6 GPa (*6*–*18*), corresponding to variations in strain rate of >10 orders of magnitude. This disagreement is generally attributed to the difficulty in making these measurements at the high stresses required for practical laboratory strain rates (typically ~10^−5^ s^−1^) to be achieved. Most minerals are brittle if deformed at these stresses, so fracture is often suppressed by increasing the confining pressure. The technical challenges associated with apparatuses for high-pressure experiments, however, result in less reliable measurements of load and displacement than in unconfined experiments.

Furthermore, laboratory predictions of olivine strength at low temperature are in conflict with estimates of the strength of the lithosphere from geodynamic simulations and geophysical observations. For example, convection simulations that incorporate plastic yield exhibit behavior similar to plate tectonics only if the yield stress is ~200 MPa (*19*–*21*). Similarly, observations of lithospheric flexure around Hawaii can only be reasonably modeled if the maximum stress supported by the lithospheric mantle is ~200 MPa (*2*). In contrast, predictions of the maximum strength of the lithosphere from most previous laboratory studies are on the order of 1 GPa (*6*, *8*, *10*).

To improve upon previous experimental work, we examined low-temperature plasticity in olivine using nanoindentation, a technique that generates a confining pressure in the sample, allowing plastic behavior to be accessed even at room temperature. Yield stress can be calculated from indentation experiments through its relation to hardness (the ratio of applied load to the projected area of the indent) (*22*). Early work on olivine by Evans and Goetze (*6*) used a four-sided pyramidal (Vickers) indenter in a dead-weight microindentation system and measured the size of the residual indent to calculate hardness and yield stress. A more recent approach by Kranjc *et al*. (*16*) used nanoindentation and a three-sided pyramidal (Berkovich) indenter tip, which relies on a calibrated area function to determine the mechanical properties of the indented material. Because of the sharpness of the indenter tips in these types of experiments, materials yield plastically almost immediately upon application of load with little preceding elastic deformation, and the stress state beneath the tip is very complex. Therefore, while we performed some Berkovich indents to directly compare with previous olivine indentation studies (*6*, *16*), we predominantly used spherical indenter tips to examine the plastic behavior of olivine.

Spherical nanoindentation has several significant benefits over previously used methods. First, the elastic-plastic transition is well defined, especially for hard materials like olivine. Second, the contact between a sphere and a flat surface is described by simple analytical solutions for the elastic stress state beneath the sphere (*23*). The stress at the elastic-plastic transition (the yield stress) can therefore be easily determined without relying on instrument calibrations. Finally, hardness can be calculated as a function of strain for the entire test, revealing mechanical behavior comparable to that seen in stress-strain curves for large-scale tests (*24*).

## RESULTS

**Fig. 1:**
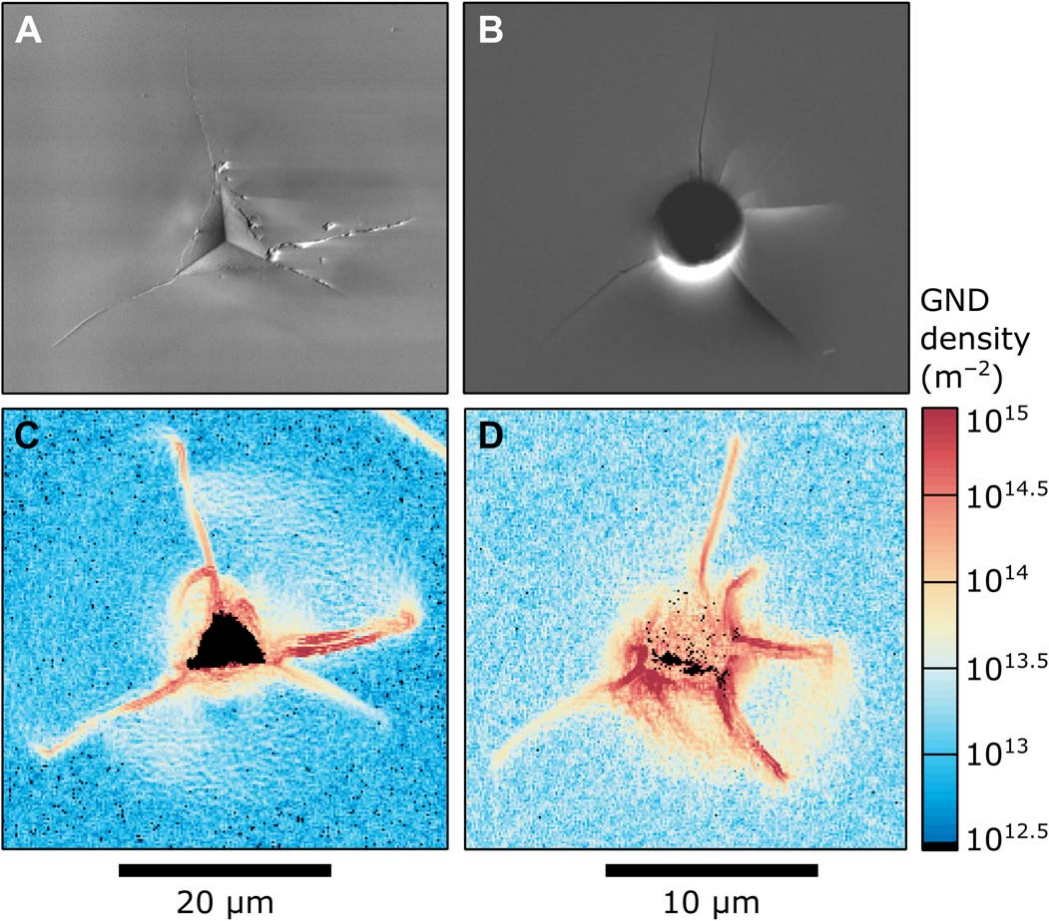
Examples of Berkovich and spherical indents. (**A**) Secondary electron image of a Berkovich indent. (**B**) Forescattered electron image of a spherical indent. (**C** and **D**) Maps of the GND densities associated with each indent as measured by HR-EBSD (*25*). The activation of plastic deformation mechanisms is shown by the elevated densities of GNDs around the residual indents. Radial fractures emanating from the indents result in artificially high GND densities in the immediate vicinity of these cracks. Regions of each map outside the plastic zone of the indents reveal the minimum resolvable dislocation density by HR-EBSD, which varies for each map based on the analytical conditions and crystal orientation.

We performed over 800 room temperature nanoindentation experiments on both single crystal (Fig. 1 and tables S1 and S2) and polycrystalline olivine (fig. S1 and table S1). Experiments in which the indenter/sample contact is not entirely elastic leave residual indents, evident in both electron micrographs (Fig. 1) and mechanical data (for example, fig. S2). Geometrically necessary dislocation (GND) densities, measured by high-angular resolution electron backscatter diffraction (HR-EBSD) (*25*), are present around the residual indents (Fig. 1), demonstrating the activation of plastic deformation mechanisms (see the Supplementary Materials for further details). Cracks are also present around the indents (Fig. 1), but sectioning by focused ion beam (FIB) milling suggests that they form during unloading and therefore do not affect the yielding behavior observed in the mechanical data (fig. S3).

**Fig. 2:**
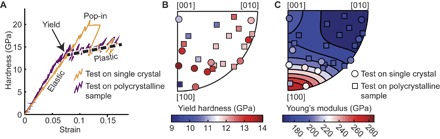
Summary of spherical indentation results. (**A**) Sample hardness-strain curves from tests with a 3-μm-radius indenter. The dashed black line is a linear fit to the hardness data after pop-in for the single crystal. This fit is projected back to the elastic portion of the data to calculate the yield hardness. (**B**) Inverse pole figure (IPF) representing the average hardness at yield calculated for each crystal orientation tested via spherical indentation. Each marker for a single-crystal sample represents the average of 16 tests. Each marker for the polycrystalline sample represents a single indentation test on one grain of the sample. (**C**) IPF illustrating the measured Young’s modulus for the same orientations as in (B). The background is colored by the theoretical Young’s modulus from Abramson *et al*. (*28*).

**Fig. 3:**
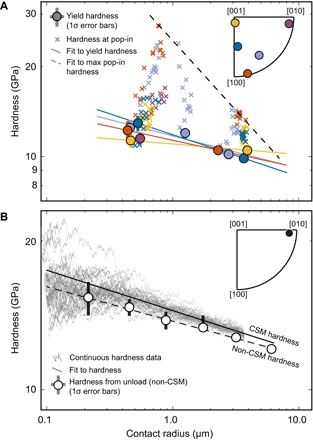
Size effects observed by spherical and Berkovich indentation. (**A**) Spherical indentation size effect for four different orientations of olivine as a function of contact radius at yield. Data for a fifth orientation (shown in purple) are also plotted but were only measured with a single indenter tip. Plotted values of yield hardness are averages over 12 to 16 tests. (**B**) Berkovich indentation size effect for a single orientation of olivine, as illustrated in the inset. Markers for non-CSM (continuous stiffness measurement) tests are averages of 6 to 10 tests.

Large “pop-in” events are sometimes present in indentation tests on single crystals and can be recognized as an abrupt increase in displacement followed by a large stress drop. These pop-in events occur at the elastic-plastic transition, as revealed in hardness-strain curves from spherical indentation tests (Fig. 2A), but the hardness at which the pop-in occurs is stochastic (Fig. 3A, fig. S4, and table S1). The observed range of hardness values at pop-in is wider for spherical indenter tips with smaller radii, and much larger hardnesses can be reached before the pop-in with these tips. Notably, pop-ins are not present in tests on polycrystalline samples of olivine that have been previously deformed at high temperature (Fig. 2A and table S1). However, the hardness after yield in the polycrystalline samples is remarkably consistent with that seen after pop-ins in single crystals.

Projection of the plastic portion of the hardness-strain curve back to the elastic portion gives an estimate of the hardness at yield (Fig. 2A and table S1). In 150 tests performed using a 3-μm-radius indenter tip across 25 crystal orientations, the average yield hardness is 12.5 ± 1.1 GPa. Although the flow behavior of olivine is anisotropic at high temperature (*26*, *27*), the yield hardness at room temperature depends very little on crystal orientation, varying less than 8% among orientations for the 10 single-crystal orientations tested (Fig. 2B). This lack of anisotropy may be due, in part, to the nature of spherical indentation, as evidenced by our observation of reduced anisotropy associated with the elastic (Young’s) modulus. The degree of elastic anisotropy measured here (variation of ~30%) is reduced relative to that measured by other methods (variation of ~45%) (*28*) due to out-of-plane forces inherent to the geometry of a spherical indentation test (Fig. 2C).

A major observation in our data set is that yield hardness varies as a function of indenter size. Tests with smaller indenters exhibit larger hardness values (Fig. 3A), a phenomenon commonly referred to as a “size effect” in the materials science literature (*29*, *30*). Measuring the magnitude of this size effect in hardness is critical for understanding how length scales of deformation can modify lithospheric strength and for properly scaling laboratory results to geological conditions. The size effect measured by spherical indentation can be characterized by a power law with an exponent of −0.09, that is, yield hardness is proportional to *a*^−0.09^, where *a* is contact radius (Fig. 3A). The maximum hardness at pop-in is also a function of contact radius (Fig. 3A), defined by a power law with a length-scale exponent of −0.47. These two power laws define an envelope bounding the size effects in the data set (Fig. 3A).

The magnitude of the size effect observed by spherical indentation is similar to that observed by Berkovich indentation (Fig. 3B). In the latter, the indented material yields almost immediately upon loading, and hardness decreases while the effective contact radius increases over the course of the test. Changes in hardness with increasing contact radius during the loading portion of the tests give a size effect exponent of −0.08. Hardness values obtained from unloading curves (table S2) in multiple tests have a similar dependence on contact radius with an exponent of −0.07. This similarity in the magnitudes of the size effects observed in spherical indentation and Berkovich indentation is especially striking considering that these are two very different types of experiment with different data processing methods. The magnitude of the size effect observed in olivine is broadly similar to those reported for industrial ceramics (*31*).

## DISCUSSION

Overall, we observe two different size effects in our data: (i) variations in the presence and maximum hardness of pop-ins (Figs. 2A and 3A) and (ii) variations in hardness at yield (Fig. 3), both as functions of the size of the indenter contact (contact radius). The presence of pop-ins in relatively pristine single crystals, and the associated lack of pop-ins in samples with grain boundaries and dislocation substructures (fig. S5), is consistent with observations in metals and ceramics, where pop-ins are interpreted as bursts of dislocation nucleation and motion (*32*). The predeformed polycrystalline sample has abundant dislocation sources; therefore, grains in this sample yield at a stress consistent with the activation of those sources. The single crystals used here, however, have a lower initial density of dislocation sources. Therefore, some indents must proceed to greater depths and larger average stresses in order for the stressed region to be large enough to activate enough dislocation sources. These effects can be mitigated by using a larger indenter, which markedly reduces the maximum pop-in hardness relative to the yield hardness (Fig. 3A).

In the second size effect, the yield hardness is directly related to the volume of the plastically deformed region, even when abundant dislocation sources are present. The size effects observed in both spherical and Berkovich indentation tests on olivine are of the same order as those seen in indentation tests on ceramics (*33*). This “smaller is stronger” phenomenon has been previously explained as a result of the role of strain gradients and associated GNDs in modifying plastic behavior (*34*, *35*). For the same total strain, smaller indenters create a smaller deformed region in which to store GNDs. Thus, the GND density in the plastically deforming region is likely higher for smaller indents, increasing the hardness of the material by limiting dislocation motion through both short- and long-range interactions. The Hall-Petch effect, in which a material with a very small grain size exhibits higher flow strength than one with a larger grain size, is thought to arise from the same source (*36*): In smaller grains, higher strain gradients form for the same strain, and dislocations associated with these strain gradients interfere with further dislocation motion and deformation. Thus, when low-temperature plasticity is the dominant deformation mechanism, the strength of polycrystalline olivine should also increase with decreasing grain size, likely following a power law similar to that observed in indentation tests. Although the commonly cited inverse square law associated with the Hall-Petch effect has a larger power-law exponent than that determined by the current study, previous work has evidenced a wide range of exponents for different materials. A general scaling relationship describing Hall-Petch–like behavior is still a subject of active debate [for example, in the study of Dunstan and Bushby (*37*)].

**Fig. 4:**
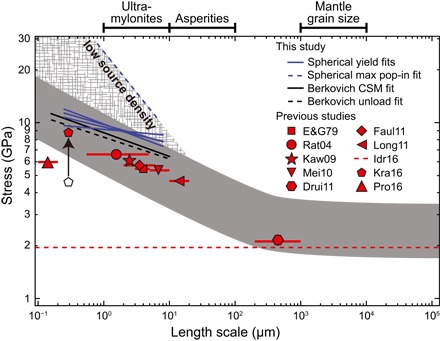
Comparison between observed size effects in our indentation tests and previously published low-temperature plasticity flow laws extrapolated to room temperature. Yield stresses from flow laws are calculated for a strain rate of 0.01 s^−1^ and a confining pressure of 3 GPa to approximately match the strain rate and confining pressure of indentation tests. Data from Druiventak *et al*. (*11*) are not from a flow law but are approximate yield stresses from experiments obtained at room temperature and confining pressures of 2.0 to 2.5 GPa. These yield stresses likely reflect plasticity before any brittle deformation. Flow laws from past indentation studies (*6*, *16*), as well as indentation data from this study, are plotted using contact radius as the length scale, whereas flow laws based on tests on polycrystalline samples (*8*–*13*, *18*) are plotted using grain size. The predicted yield stress from Idrissi *et al*. (*17*), in which dislocation velocity was measured to calibrate a flow law, is assumed to be independent of scale. The predicted yield stress from Kranjc *et al*. (*16*) has been rescaled following the method used by Evans and Goetze (*6*) to calculate the constraint factor (the original data point is plotted as an open pentagon). This rescaling has been performed so that all estimates from indentation experiments using sharp tips are processed in the same manner. The dark gray band represents the approximate size effect observed in all data, with the hatched gray field indicating even larger yield stresses when the dislocation source density is low. Relevant geological length scales are indicated (*38*, *39*, *41*–*45*). The predicted stress from Demouchy *et al*. (*14*) is not included because their flow law is calibrated using the maximum stress observed in experiments rather than the yield stress.

Size effects have not been considered in previous studies of low-temperature plasticity in olivine. However, most previous experiments were likely affected by size effects because they were either (i) conducted using indentation techniques with inherently small regions of plastic deformation or (ii) conducted on polycrystalline aggregates with small grain sizes. Therefore, we suggest that size effects constitute the major source of disagreement among published results. We compare our measured size effects with flow laws from previous studies extrapolated to room temperature in Fig. 4, after first converting hardness values from our indentation data to yield stresses. Although it is difficult to directly compare experiments with different definitions of length scale, this analysis of different flow laws reveals a similar power-law relationship between length scale (grain size for polycrystalline deformation and contact radius for indentation tests) and room temperature strength as observed in our indentation tests, demonstrating that a size effect can account for most of the discrepancy in published data.

The observed size effect in yield stress suggests that data from samples with large inherent length scales (*11*, *17*) best represent the plastic strength of olivine in the coarse-grained lithospheric mantle (*38*, *39*). Thus, we suggest that the flow law from Idrissi *et al*. (*17*) is the best available for capturing the strength of coarse-grained mantle at low temperatures (Fig. 4) and indicates that the lithospheric mantle is weak relative to previous experimental predictions. This particular flow law is based on the incorporation of measurements of dislocation velocity into a micromechanical simulation, which does not involve any inherent size effects. Notably, it predicts a maximum strength of the lithosphere on the order of hundreds of megapascals, in much better agreement with geodynamic and geophysical estimates of lithospheric strength than with most published flow laws (*6*, *8*–*10*, *12*, *13*, *16*).

Another major geophysical implication of the size effect in olivine is that fine-grained peridotite aggregates deforming by low-temperature plasticity will be stronger than their coarse-grained equivalents. This behavior is opposite to that observed in tests on polycrystalline olivine at higher temperatures, which activate deformation mechanisms for which strength increases with increasing grain size (*40*). The compilation of published olivine plasticity data in Fig. 4 allows us to estimate the length scale below which decreasing grain size will strengthen olivine. This critical length scale is approximately 300 μm, which is comparable to or larger than grain sizes in exhumed mylonitic mantle shear zones (*41*–*43*). In shear zones that localize strain due to grain size reduction during deformation at high temperatures, low-temperature plasticity will be a relatively strong mechanism. Thus, later deformation at low temperatures will more likely occur via other mechanisms, such as brittle fracture.

Finally, a size effect has important consequences for the deformation and evolution of olivine-rich faults in mantle rocks. Experimental rate-and-state friction tests on most geologic materials reveal a “critical slip distance,” which is commonly interpreted as the average asperity size (Fig. 4), on the order of tens of micrometers (*44*). In addition, experiments have suggested that contact pressures on asperities are large enough to induce plastic deformation, even for harder geologic materials such as olivine (*45*). Because plastic deformation of microscopic asperities on faults is akin to that which occurs in an indentation test, the size effect seen in our experiments will also exist on faults (that is, asperity strength will increase with decreasing asperity size). For a distribution of asperity sizes, the presence of a size effect could result in intrinsic heterogeneity in the mechanical and frictional properties of faults, which may control the evolution of fault roughness. Our results are also consistent with predictions from previous work on fault roughness (*46*), which suggests a link between length scale and strength.

Although much work remains to be done in characterizing size effects across the range of geologically relevant materials, this study demonstrates their impact on a wide range of geodynamic phenomena involving deformation of the lithosphere.

## MATERIALS AND METHODS

Nanoindentation experiments with a spheroconical diamond indenter tip were performed using an MTS Nanoindenter XP on polished olivine samples. Tips with different radii were used to measure indentation size effects. Individual tests recorded load, displacement, contact stiffness, and time. Using the equations of Kalidindi and Pathak (*47*), we calculated the hardness and contact radius at yield, which was then converted to yield stress by applying a constraint factor.

Nanoindentation experiments with a diamond Berkovich tip were performed using an iMicro Nanoindenter (Nanomechanics Inc.). As with the spherical tests, individual Berkovich experiments record load, displacement, contact stiffness, and time. Hardness and elastic modulus were determined using the unloading method of Oliver and Pharr (*48*) or using the continuous stiffness method (*49*). The constraint factor was calculated following the equations laid out by Evans and Goetze (*6*).

The distribution of GNDs was measured using HR-EBSD, performed on a FEI Quanta 650 FEG ESEM. This technique uses cross-correlation of regions of interest in electron diffraction patterns to resolve changes in crystal orientation on the order of ~0.01° (*25*).

A three-dimensional analysis of the crack structure formed around residual spherical indents was performed using AURIGA CrossBeam Workstation with a GEMINI FE-SEM Column to progressively mill into several indents.

More details on materials and methods are available in the Supplementary Materials.

## Supplementary Material

http://advances.sciencemag.org/cgi/content/full/3/9/e1701338/DC1

## SUPPLEMENTARY MATERIALS

Supplementary material for this article is available at http://advances.sciencemag.org/cgi/content/full/3/9/e1701338/DC1 Supplementary Materials and Methods fig. S1. EBSD map of the 120-indent array on PI-1488. fig. S2. Zero-point correction for spherical indentation. fig. S3. Creation of new surface crack due to stress release from FIB milling. fig. S4. Four groups of hardness-strain curves proceeding to different total strains on sample OP4-2. fig. S5. EBSD map of a portion of PI-1488, colored by GND density. table S1. Summary of deepest spherical indentation tests. table S2. Summary of non-CSM Berkovich indentation tests. References (*50*–*59*)
