# Supplementary material for: Size effects resolve discrepancies in 40 years of work on low-temperature plasticity in olivine
Source: Sci Adv. 2017 Sep 13;3(9):e1701338. doi: 10.1126/sciadv.1701338 (PMC5597306; doi:10.1126/sciadv.1701338)
Supplement: http://advances.sciencemag.org/cgi/content/full/3/9/e1701338/DC1 [file supp_3_9_e1701338__index.html]

Science Advances | Science Advances

## Supplementary Materials

**This PDF file includes:**

- Supplementary Materials and Methods
- fig. S1. EBSD map of the 120-indent array on PI-1488.
- fig. S2. Zero-point correction for spherical indentation.
- fig. S3. Creation of new surface crack due to stress release from FIB milling.
- fig. S4. Four groups of hardness-strain curves proceeding to different total strains on sample OP4-2.
- fig. S5. EBSD map of a portion of PI-1488, colored by GND density.
- Legends for tables S1 and S2
- References (*50–59*)

Download PDF

**Other Supplementary Material for this manuscript includes the following:**

- table S1 (Microsoft Excel format). Summary of deepest spherical indentation tests.
- table S2 (Microsoft Excel format). Summary of non-CSM Berkovich indentation tests.

**Files in this Data Supplement:**

- Adobe PDF - 1701338\_SM.pdf
