# Supplementary material for: Size effects resolve discrepancies in 40 years of work on low-temperature plasticity in olivine
Source: Sci Adv. 2017 Sep 13;3(9):e1701338. doi: 10.1126/sciadv.1701338 (PMC5597306; doi:10.1126/sciadv.1701338)
Supplement: http://advances.sciencemag.org/cgi/content/full/3/9/e1701338/DC1 [file 1701338_SM.pdf]

Science Advances

AAAS

advances.sciencemag.org/cgi/content/full/3/9/e1701338/DC1

Supplementary Materials for

Size effects resolve discrepancies in 40 years of work on low-temperature plasticity in olivine

Kathryn M. Kumamoto, Christopher A. Thom, David Wallis, Lars N. Hansen, David E. J. Armstrong, Jessica M. Warren, David L. Goldsby, Angus J. Wilkinson

Published 13 September 2017, *Sci. Adv.* **3**, e1701338 (2017)
DOI: 10.1126/sciadv.1701338

The PDF file includes:

- Supplementary Materials and Methods
- fig. S1. EBSD map of the 120-indent array on PI-1488.
- fig. S2. Zero-point correction for spherical indentation.
- fig. S3. Creation of new surface crack due to stress release from FIB milling.
- fig. S4. Four groups of hardness-strain curves proceeding to different total strains on sample OP4-2.
- fig. S5. EBSD map of a portion of PI-1488, colored by GND density.
- Legends for tables S1 and S2
- References (*50*–*59*)

Other Supplementary Material for this manuscript includes the following:
(available at advances.sciencemag.org/cgi/content/full/3/9/e1701338/DC1)

- table S1 (Microsoft Excel format). Summary of deepest spherical indentation tests.
- table S2 (Microsoft Excel format). Summary of non-CSM Berkovich indentation tests.

SUPPLEMENTARY MATERIALS AND METHODS

Sample materials and preparation

Samples were cut from single crystals of olivine to produce specimens with two flat, parallel sides. One side of each crystal section was polished using a combination of high-grit SiC sandpaper and diamond solutions down to a grit size of 0.06 μm. For indentation, sections were mounted on an aluminum stub with the polished side up. Crystal orientations were determined with electron backscatter diffraction (EBSD) using the FEI Quanta 650 FEG E-SEM in the Department of Earth Sciences, University of Oxford, equipped with an Oxford Instruments Aztec 2.1 EBSD system and NordlysNano EBSD camera.

In order to examine the role of pre-existing dislocations on the deformation behavior of olivine, we also prepared a previously deformed polycrystalline aggregate of olivine for indentation. Sample PI-1488 was shortened to 5% longitudinal strain at ~1400 K and a confining pressure of 300 MPa in a Paterson apparatus. The average grain size of the sample is 9.6 μm. Deformation of this sample was originally reported in Hansen et al. (*40*).

Spherical indentation data acquisition

Spherical indentation tests were performed using an MTS XP Nanoindenter and spheroconical diamond indenter tips ranging in radius from 3 to 19 μm. Nominal strain rate, defined as the loading rate divided by the load, was controlled at 0.05 s^{-1}. Indentation strain rates, calculated using the definition of strain given below, ranged from $7x10^{-4}$ to $2x10^{-3}$ s^{-1} over the course of a single test. Individual tests record load (P), displacement (h), contact stiffness (S), and time (t).

All indenter tips were first tested on a fused silica standard to determine the effective radius of the tip. Fused silica is a common standard in nanoindentation, and its behavior is well-defined and isotropic (*48*). We performed 16 purely elastic indents (h = 40 nm for the smallest indenter tip, h = 500 nm for the largest indenter tip) in fused silica. The effective modulus of the test (E_{eff}) can be calculated from the known Young's modulus (E) and Poisson's ratio (v) of diamond and fused silica according to

$$\frac{1}{E_{\text{eff}}} = \frac{1 - v_{\text{s}}^2}{E_{\text{s}}} + \frac{1 - v_{\text{i}}^2}{E_{\text{i}}} \tag{1}$$

where the subscripts s and i refer to the sample and the indenter tip, respectively (*50*). The effective radius of the indenter (R_{eff}) can then be calculated using the well-known relationship for a Hertzian contact (*50*)

$$P = \frac{4}{3} E_{\text{eff}} R_{\text{eff}}^{1/2} h^{3/2} \tag{2}$$

Because these calibration indents were purely elastic, R_{eff} is taken to be equal to the true indenter radius.

Indentation tests on olivine were carried out to specified depths of 70 nm, 300 nm, 600 nm, and 800 nm for all single crystals using a 3 µm radius tip with 16 tests at each depth. Each test was spaced at least 50 µm away from other indents. Tests were also run using a 7 µm and a 19 µm indenter on several samples to examine the effect of indenter radius on indentation behavior.

Tests with the 7 µm indenter went to depths of 100 nm and 1000 nm. Tests with the 19 µm indenter went to depths of 500 nm and 600 nm. Once the specified depth was reached in each test, the load was held constant for 10 seconds to measure thermal drift. The sample was then unloaded to 10% of maximum load and subjected to another 10-second hold, also to measure thermal drift.

For the polycrystalline sample (PI-1488), rather than attempt to locate individual indents within specific grains, we instead created a large array of 120 indents over an approximately 580 x 150 µm area of the sample. Each indent proceeded to a depth of 600 nm. After indentation, an EBSD map of the same area was collected with a step size of 0.5 µm to identify the location of the indents relative to grain boundaries (fig. S1). Of the 120 indents performed, 15 were determined to be sufficiently far from grain boundaries to reasonably characterize the behavior of grain interiors due to the small size of the indentation test relative to the grain size. Note that we are unable to identify grain boundaries below the sample surface using this technique.

Since deformation in nanoindentation is computed relative to the initial position of the surface of the sample, accurately determining the point at which the indenter tip first contacts the surface is critical for accurately measuring loads and displacements during the test. Since the advent of continuous stiffness measurements (CSM) in nanoindentation, the touch point is usually assumed to be the point at which the measured stiffness first reaches 200 N/m. Imperfections of the sample surface and of the indenter tip, however, can lead to erroneous early surface detection, particularly for spherical indentation, wherein an early surface detection produces a large spike in plots of hardness versus strain.

Spherical indentation data processing

Kalidindi and Pathak (*47*) proposed a method for determining the effective zero point for spherical indentation. This method evaluates the region over which the indentation behavior measured by the instrument matches the theoretical elastic behavior predicted by Hertz's theory (Eq. 2). Data before the elastic section are removed, while data after the elastic section are assumed to represent permanent deformation. Although the effective zero point may not exactly coincide with the true zero point, the correction procedure effectively removes artifacts that have significant effects on calculations of the early elastic behavior of the sample (fig. S2).

After this zero-point correction, E_{eff} is calculated for each crystal by fitting data from the purely elastic 70 nm depth tests using the 3 µm indenter to Eq. 2. E_s is then calculated following Eq. 1. Data from tests with larger indenter tips on sample OP4-2 were found to exhibit the same E_s as those from tests using the 3 µm radius tip. Therefore, the moduli calculated from the 70 nm tests with the 3 µm indenter are taken to be representative of tests with larger indenter radii on the same crystals. For the polycrystalline sample, E_{eff} is calculated by individually fitting Eq. 2 to the elastic portion of the data in each test before yield.

We use Kalidindi and Pathak's (*47*) proposed definitions for contact radius (a) and strain (ε), taking advantage of CSM to calculate contact radius, hardness (H), and strain at all points in the test. This approach is a major improvement over previous methods, in which only the unload curve was used and each individual indentation test could only produce one data point on a hardness-strain curve. We calculate hardness and strain following their methodology using the following equations

$$a = \frac{S}{2E_{\text{eff}}} \quad (3)$$

$$H = \frac{P}{\pi a^2} \quad (4)$$

$$\varepsilon = \frac{4}{3\pi}\frac{h}{a} \quad (5)$$

For tests that exhibited a pop-in, the hardness at "yield" is calculated by linearly projecting the data characterizing plastic flow back to the elastic portion of the hardness-strain curve (Fig. 2A). The intersection between the elastic portion of the test and the projection of the flow portion of the test is reproducible for each crystal orientation and, in tests with a pop-in, is assumed to represent the elastic-plastic transition for a crystal of the same orientation with more abundant dislocation sources.

The hardness is larger than the differential stress (σ_{d}) of the material being indented due to the inherent confining nature of the experiment. However, H and σ_{d} are directly proportional via a constant known as the constraint factor (C)

$$H = C\sigma_d \quad (6)$$

The constraint factor asymptotically approaches ~3 for sharp tip geometries (*22*). At the elastic-plastic transition in spherical indentation, however, the constraint factor can be quite minimal (*23*, *51*). Following a finite-element analysis of spherical indentation conducted by Donohue *et al.* (*52*), we divide our hardness values at the elastic-plastic transition by a constraint factor of 1.2 to extract yield stress.

Berkovich indentation data acquisition and processing

Berkovich indentation tests were performed using a diamond tip in an iMicro Nanoindenter (Nanomechanics, Inc., Oak Ridge, TN) on a single crystal of olivine oriented 14˚ off the [010] axis. Nominal strain rate, again defined as the loading rate divided by the load, was maintained at 0.2 s^{-1}. Constant strain rate experiments consisted of an exponential loading phase to a peak load of 400 mN. Following a 5-s hold at peak load, the sample was unloaded to 10% of the peak load. A 90-s hold was then imposed to measure the thermal drift rate prior to the final unloading sequence to bring the tip out of contact with the sample. In all tests, the azimuthal orientation of the sample relative to the indenter tip was fixed, eliminating all possible orientation effects. We used the continuous stiffness measurement (CSM) method to measure contact stiffness throughout the loading phase of the experiment. By continuously measuring stiffness, E_{eff} and H are determined as a function of depth in an experiment (*49*).

Some experiments were performed without CSM, with stiffness determined from the unloading curve using the Oliver-Pharr method (*48*). Peak loads ranging from 3 mN to 1 N were imposed for a series of indents to investigate the depth (size) dependence of hardness (table S2).
Constraint factors for converting Berkovich hardness values to yield stresses were calculated using the method of Evans and Goetze (*6*)

$$C = \frac{H}{\sigma_{\mathrm{y}}} = 0.19 + 1.6 \log \frac{E \tan \beta}{\sigma_{\mathrm{y}}} \tag{7}$$

where β is the effective cone angle of the indenter (70.3˚ for Berkovich geometry). Larger ratios of Young's modulus (E) to yield stress (σ_{y}) result in larger values of C that asymptotically approach ~3.0 in the fully plastic regime. At small values of E/σ_{y}, the constraint factor

approaches ~1.0. The constraint factors for the Berkovich data range from 1.4 and 1.8, well within the intermediate elastic-plastic transition regime described by Johnson (*51*).

The self-similar geometry of the Berkovich indenter can be approximated as a cone for the purposes of calculating contact radius. For a conical indenter, the effective contact radius is related to the indentation depth by the tangent of the cone angle

$$a = h\tan(\beta) \tag{8}$$

High-angular resolution electron backscatter diffraction

To map distributions of geometrically necessary dislocations (GNDs) we employed high-angular resolution electron backscatter diffraction (HR-EBSD) (*25*, *53*, *54*). For HR-EBSD, diffraction patterns are captured in the same manner as for conventional EBSD, but are stored for HR-EBSD post-processing. EBSD data were collected using the same FEI Quanta 650 FEG E-SEM detailed previously. A map of 210 x 210 data points, at a step size of 100 nm, was collected across a residual spherical indent from a test that reached a maximum depth of 800 nm in sample OP4-2 (Fig. 1). A map of 250 x 250 data points, at a step size of 200 nm, was collected across a residual Berkovich indent (Fig. 1). Six additional maps were collected on other indents with similar HR-EBSD setups. Diffraction patterns were saved at the full resolution of the EBSD camera, i.e. 1344 x 1024 pixels, as 8-bit .tiff files.

An additional map of 850 x 575 data points, at a step size of 200 nm, was collected from polycrystalline sample PI-1488 (fig. S5), which was previously shortened to 5% longitudinal strain at ~1400 K, and initially characterized by Hansen *et al*. (*40*). Pixels in the diffraction

patterns from this map were binned 2 x 2, so that patterns were saved at a resolution of 672 x 512 pixels. This map was collected prior to indentation.

The HR-EBSD technique was developed for materials science applications (*53*, *54*) and has been recently adapted and optimized to analyze olivine (*25*). Estimates of densities and types of GNDs are highly sensitive to the precision in measurement of the axis and angle of misorientation between points in the lattice (*25*, *54*, *55*). Conventional EBSD uses Hough transforms of diffraction patterns to index crystal orientations, giving precision of 0.1–1.0° in measurements of misorientation angles and relatively poor definition of misorientation axes if the angles are small (*54*–*56*). In contrast, HR-EBSD employs cross-correlation of stored diffraction patterns to determine the displacement gradient tensor (i.e., the elastic distortion, $\boldsymbol{\beta}^{e}$) at each point in an EBSD map. This distortion can be split into symmetric and antisymmetric parts representing elastic strains and rotations of the crystal lattice, respectively (*53*). This method enables measurement of gradients in lattice orientation with angular precision of approximately 0.01° and well-defined misorientation axes (*53*, *57*). Following Wilkinson and Randman (*54*), the dislocation tensor ($\boldsymbol{\alpha}$) of Nye (*58*) is related to the distortion field by

$$\boldsymbol{\alpha} = curl(\boldsymbol{\beta}^{e}) \tag{9}$$

where the elements of α_{ij} are related to the densities (ρ_s) of s dislocation types, with Burgers vectors b^s and line directions l^s, through

$$\alpha_{ij} = \sum_{s=1}^{s_{\max}} \rho_s b_i^s l_j^s \tag{10}$$

This approach assumes that the elastic strain components of $\boldsymbol{\alpha}$ are negligible compared to the lattice rotations, which is the case in our maps. The opening of cracks present at the indent sites will contribute to an elevated apparent GND density for patches of material spanning the cracks. We follow the method detailed by Wallis *et al.* (*25*) to calculate a single best-fit solution for densities of six common types of GNDs in olivine from the six components of lattice rotation measurable from 2-D HR-EBSD maps. Summation of the densities of all dislocations types at each point generates a map of the total dislocation density. Estimates of GND density obtained from 2-D maps provide a lower bound on the total GND density in 3-D. As the noise level in estimates of GND density is proportional to the precision of the measurements of lattice curvature, the use of HR-EBSD allows us to resolve significantly lower densities of GNDs (~10^{13} m^{-2}) than would be achievable using conventional EBSD (>10^{14} m^{-2}) (*25*, *54*). Regions of each map outside the plastic zone of the indents reveal the minimum detectible dislocation density at the given conditions.

Focused ion beam milling

The structure of cracks around residual indents can be used to infer the circumstances under which the cracks formed. To analyze the morphology of these cracks, we performed milling with a focused ion beam (FIB) on several indents. After indentation tests were completed, the sample was coated with 10 nm of platinum before being placed in an AURIGA CrossBeam Workstation with a GEMINI FE-SEM Column. Successive trenches were milled using a gallium ion beam with a current of 1 nA.

SUPPLEMENTARY INFORMATION

Spherical indentation results

Tests were performed at 4 different depths on each single crystal. On any given crystal, data were reproducible, exhibiting similar ranges of hardness at a given strain (fig. S4). Therefore, only the deepest tests are used in all the plots. These data are summarized in table S1. Because the indentation apparatus has a maximum load of 500 mN, some tests implementing the 19 μm indenter did not yield or included relatively few data points after yield prior to reaching the maximum load. In these cases, maximum hardness is reported as a pop-in, and no associated yield hardness is reported.

Berkovich indentation results

Data from tests conducted without CSM are summarized below in table S2. Data from tests conducted with CSM are available upon request.

HR-EBSD results

HR-EBSD results demonstrate that lattice curvature around an indent in OP4-2 corresponds to GND densities of $>10^{14}$ m^{-2} close to the rim of the residual indent (Fig. 1). The distribution of GNDs around the indent is asymmetrical due to the plastic anisotropy of olivine single crystals (Fig. 1). Densities of GNDs decrease with distance from the indent to a background noise level of $<10^{13.5}$ m^{-2} observed in the portion of the crystal unaffected by the indent (Fig. 1). Wallis *et al*. (*25*) determined that the dislocation density of the undeformed starting material is approximately 10^{10} m^{-2}. Prominent radial bands of *apparent* high GND density result from small rotations across microcracks evident in the secondary electron image (Fig. 1). The map of GND density

(Fig. 1) demonstrates that nanoindentation introduced high densities of GNDs that are significantly elevated above the background level and therefore were not present prior to nanoindentation. Other HR-EBSD maps of indents show very similar patterns of GNDs.

HR-EBSD results from the polycrystalline deformed sample, PI-1488, reveal a heterogeneous distribution of GNDs at relatively high densities (fig. S5). GND densities generally exceed 10^{14} m^{-2} and locally exceed 10^{15} m^{-2}, with the highest densities commonly located close to grain boundaries. Typically, GNDs are relatively smoothly distributed, reflecting continuous lattice curvature, although discrete bands of locally elevated GND density are present in some grains, corresponding to the presence of subgrain boundaries. Individual grains with anomalously high apparent GND densities of $>10^{15.5}$ m^{-2} are in poor orientations for resolving the GND content and so have exceptionally high noise-floors.

Fracture structure around indents

Surface-breaking fractures were often seen to start from the edge of the final indent impression and thus were not present earlier in the experiment when the contact radius was smaller, suggesting they do not form during the loading phase (fig. S3). Additionally, observation of the fractures during FIB milling showed additional radial surface cracks being created as residual stresses were released due to the removal of material (fig. S3). We therefore conclude that the cracks must have formed during or after the unloading process. Theoretical modeling of a spherical indent shows that hoop stresses around the indent are compressional during loading, supporting the conclusion that radial cracks could not form in the loading phase of the experiment (Figure 5.4.3b in *59*).

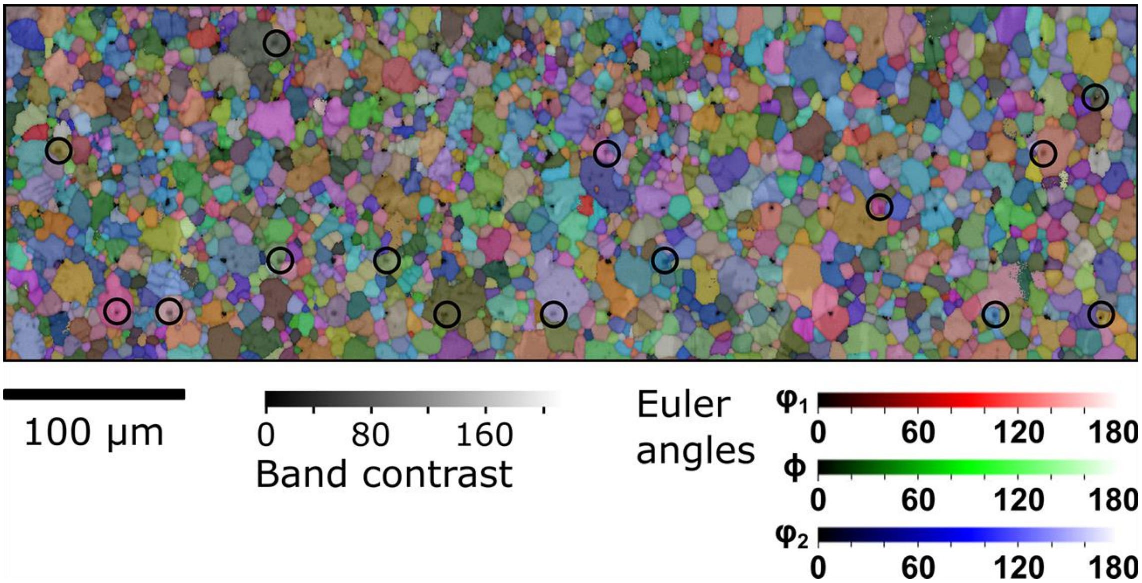

fig. S1. EBSD map of the 120-indent array on PI-1488. Grains are colored by Euler angle and shaded by band contrast. Indents generally appear as dark dots in band contrast. The indents used for calculations are circled in black.

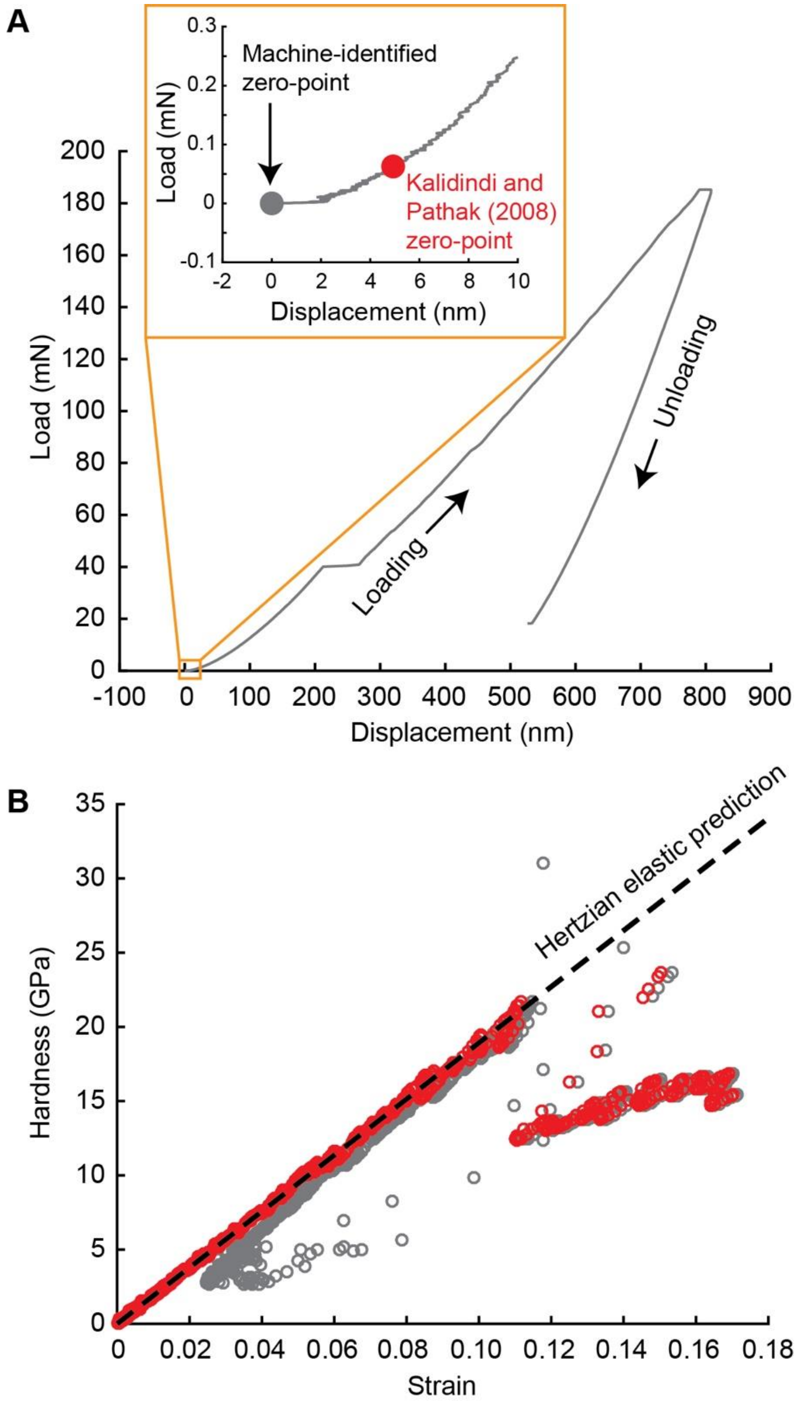

fig. S2. Zero-point correction for spherical indentation. (A) A typical load-displacement curve for a spherical indent in olivine using the 3 µm indenter. Note the offset in displacement between the start of the loading curve and the end of the unloading curve. This represents the permanent deformation associated with the residual indent. In the inset, the zero-point identified

by the MTS XP Nanoindenter is in grey, and the zero-point identified by the method of Kalidindi and Pathak (*47*) is in red. **(B)** The resulting hardness-strain curves assuming the Kalidindi and Pathak (*47*) zero point (red) versus the machine-identified zero point (grey).

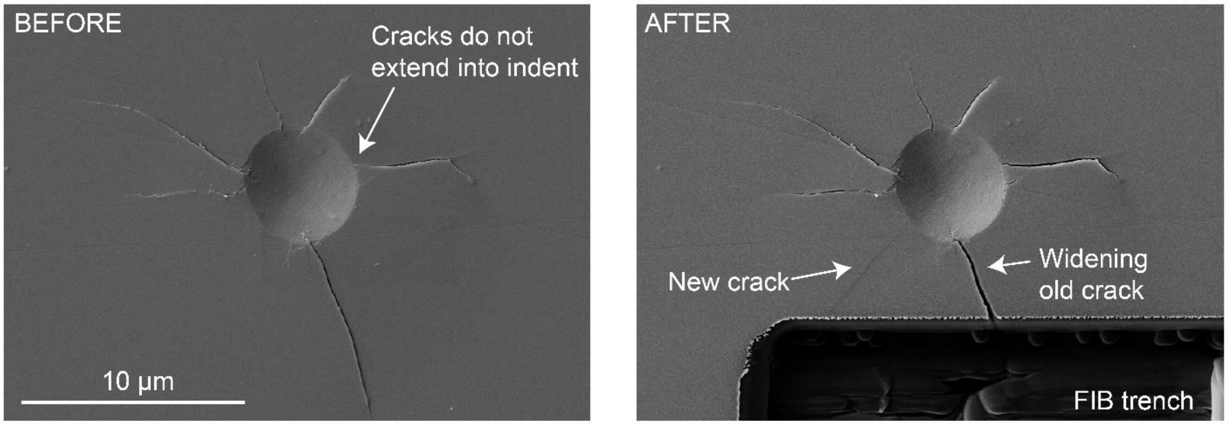

fig. S3. Creation of new surface crack due to stress release from FIB milling. Before and after images shown. The milling-induced fracture supports the theory that the radial cracks form due to stress release after the indent has been formed. Additional support is provided by the fact that the radial cracks do not extend into the indent itself, suggesting that they are created after the indent is fully formed.

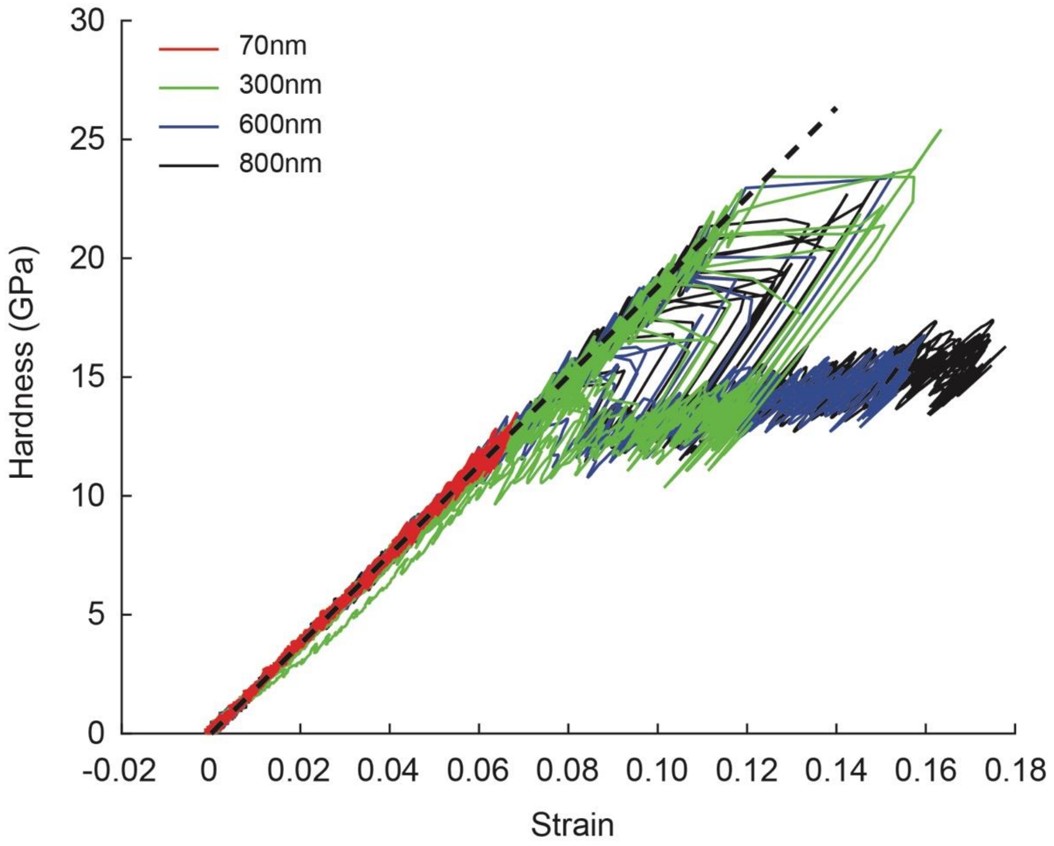

fig. S4. Four groups of hardness-strain curves proceeding to different total strains on sample OP4-2. Each color indicates a different maximum depth for the indentation test. Although the hardness at pop-in is stochastic, the elastic and plastic portions of the test are reproducible, regardless of the stress at pop-in or the final depth of the test.

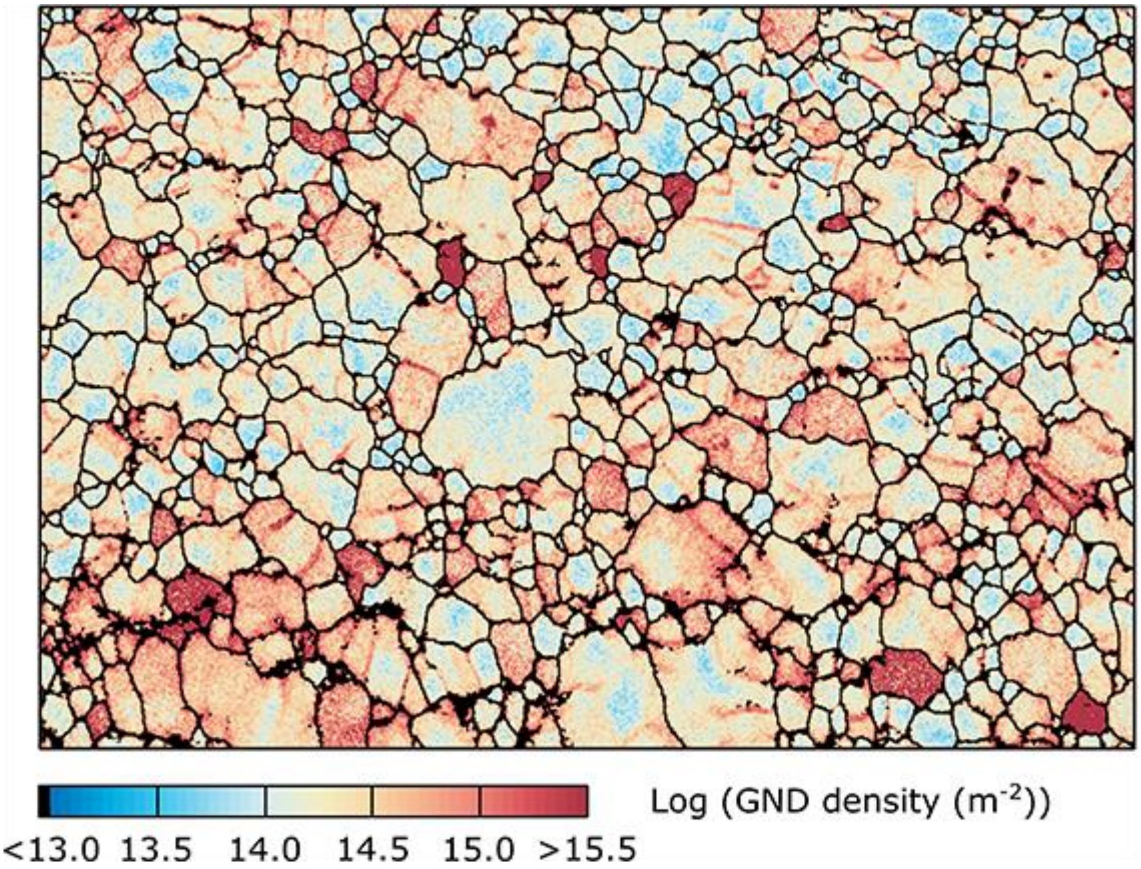

fig. S5. EBSD map of a portion of PI-1488, colored by GND density. Subgrain boundaries and grains with distributed dislocations are both present in this sample.

table S1. Summary of deepest spherical indentation tests. Young’s modulus, contact radius and hardness at yield, and contact radius and hardness at pop-in (if applicable) obtained via spherical indentation. Data from 800 nm tests are presented for single crystals, and data from 600 nm are presented for the polycrystalline sample.

table S2. Summary of non-CSM Berkovich indentation tests. Non-CSM hardness and associated contact radius calculated for Berkovich indents with different maximum loads.
